# Extracellular Vesicles Released from *Mycobacterium tuberculosis*-Infected Neutrophils Promote Macrophage Autophagy and Decrease Intracellular Mycobacterial Survival

**DOI:** 10.3389/fimmu.2018.00272

**Published:** 2018-02-19

**Authors:** Violeta D. Alvarez-Jiménez, Kahiry Leyva-Paredes, Mariano García-Martínez, Luis Vázquez-Flores, Víctor Gabriel García-Paredes, Marcia Campillo-Navarro, Israel Romo-Cruz, Víctor Hugo Rosales-García, Jessica Castañeda-Casimiro, Sirenia González-Pozos, José Manuel Hernández, Carlos Wong-Baeza, Blanca Estela García-Pérez, Vianney Ortiz-Navarrete, Sergio Estrada-Parra, Jeanet Serafín-López, Isabel Wong-Baeza, Rommel Chacón-Salinas, Iris Estrada-García

**Affiliations:** ^1^Departamento de Inmunología, Escuela Nacional de Ciencias Biológicas (ENCB), Instituto Politécnico Nacional (IPN), Mexico City, Mexico; ^2^Departamento de Fisiología y Farmacología, Facultad de Medicina Veterinaria y Zootecnia, Universidad Nacional Autónoma de México (UNAM), Mexico City, Mexico; ^3^Departamento de Biología Celular, Centro de Investigación y de Estudios Avanzados del Instituto Politécnico Nacional (CINVESTAV-IPN), Mexico City, Mexico; ^4^Laboratorio de Citometría de Flujo de Diagnóstico Molecular de Leucemias y Terapia Celular SA. De CV. (DILETEC), Mexico City, Mexico; ^5^Laboratorios Nacionales de Servicios Experimentales (LANSE), Centro de Investigación y de Estudios Avanzados del Instituto Politécnico Nacional (CINVESTAV-IPN), Mexico City, Mexico; ^6^Departamento de Bioquímica, Escuela Nacional de Ciencias Biológicas (ENCB), Instituto Politécnico Nacional (IPN), Mexico City, Mexico; ^7^Departamento de Biomedicina Molecular, Centro de Investigación y de Estudios Avanzados del Instituto Politécnico Nacional (CINVESTAV-IPN), Mexico City, Mexico; ^8^Unidad de Desarrollo e Investigación en Bioprocesos (UDIBI), Escuela Nacional de Ciencias Biológicas (ENCB), Instituto Politécnico Nacional (IPN), Mexico City, Mexico

**Keywords:** extracellular vesicles, neutrophils, tuberculosis, macrophage, autophagy

## Abstract

Tuberculosis is an infectious disease caused by *Mycobacterium tuberculosis* (Mtb). In the lungs, macrophages and neutrophils are the first immune cells that have contact with the infecting mycobacteria. Neutrophils are phagocytic cells that kill microorganisms through several mechanisms, which include the lytic enzymes and antimicrobial peptides that are found in their lysosomes, and the production of reactive oxygen species. Neutrophils also release extracellular vesicles (EVs) (100–1,000 nm in diameter) to the extracellular milieu; these EVs consist of a lipid bilayer surrounding a hydrophilic core and participate in intercellular communication. We previously demonstrated that human neutrophils infected *in vitro* with Mtb H37Rv release EVs (EV-TB), but the effect of these EVs on other cells relevant for the control of Mtb infection, such as macrophages, has not been completely analyzed. In this study, we characterized the EVs produced by non-stimulated human neutrophils (EV-NS), and the EVs produced by neutrophils stimulated with an activator (PMA), a peptide derived from bacterial proteins (fMLF) or Mtb, and observed that the four EVs differed in their size. Ligands for toll-like receptor (TLR) 2/6 were detected in EV-TB, and these EVs favored a modest increase in the expression of the co-stimulatory molecules CD80, a higher expression of CD86, and the production of higher amounts of TNF-α and IL-6, and of lower amounts of TGF-β, in autologous human macrophages, compared with the other EVs. EV-TB reduced the amount of intracellular Mtb in macrophages, and increased superoxide anion production in these cells. TLR2/6 ligation and superoxide anion production are known inducers of autophagy; accordingly, we found that EV-TB induced higher expression of the autophagy-related marker LC3-II in macrophages, and the co-localization of LC3-II with Mtb inside infected macrophages. The intracellular mycobacterial load increased when autophagy was inhibited with wortmannin in these cells. In conclusion, our results demonstrate that neutrophils produce different EVs in response to diverse activators, and that EV-TB activate macrophages and promote the clearance of intracellular Mtb through early superoxide anion production and autophagy induction, which is a novel role for neutrophil-derived EVs in the immune response to Mtb.

## Introduction

Tuberculosis is an infectious disease that causes more than a million deaths per year worldwide. The infection with *Mycobacterium tuberculosis* (Mtb) is transmitted by aerosols, and macrophages are the first immune cells that have contact with Mtb in lung alveoli, through their toll-like receptors (TLRs), NOD-like receptors, and C-type lectin-like receptors (1). The binding of these receptors with their ligands induces Mtb phagocytosis and the production of pro-inflammatory cytokines, including TNF-α, IL-6, IL-8, and IL-1β, which promote activation and migration of other immune cells, such as neutrophils, to the infected site (2, 3). Neutrophils are the most abundant phagocytic cells of the innate immune system and are crucial for the immune response to Mtb, since the absence of neutrophils accelerates death in Mtb-infected mice (4). Patients with active pulmonary tuberculosis present abundant neutrophils in sputum samples and bronchoalveolar lavages, which indicates that these cells are relevant during human infection with Mtb (5). Neutrophils phagocytose Mtb and kill them in the phagolysosome, which contains several antimicrobial molecules, such as myeloperoxidase, neutral proteinases (mainly cathepsin G, elastase, and proteinase 3), bactericidal/permeability-increasing protein and defensins (6, 7). In addition, neutrophils are able to trap Mtb in neutrophil extracellular traps (NETs), although they are unable to eliminate the mycobacteria (8). Moreover, neutrophils cooperate with other cellular elements of the immune response, such as dendritic cells (DCs), which mount T cell responses to mycobacteria (9).

Neutrophils participate in several intercellular communication networks. One of these networks has attracted interest in recent years and involves the release of extracellular vesicles (EVs) to the extracellular milieu. Human neutrophil-derived EVs were first described when these cells were incubated with sublytic doses of complement (10). The EVs that are released by neutrophils are formed by a lipid bilayer with trans-membrane proteins, which limits an internal milieu with hydrophilic components; this membrane is derived from the neutrophil cellular membrane, so these EVs are classified as ectosomes. Neutrophil-derived EVs have phosphatidylserine in the outer layer of their membranes, and also contain CR1/CD35, LFA-1/CD11a, CD11b, FcγRIII/CD16, L-selectin, HLA class I, CD66b, DAF/CD55, and CD59 (11); their diameter ranges from 100 to 1,000 nm, and they participate in intercellular communication, modulating several biological processes (12). For example, neutrophil-derived EVs decrease the phagocytic capacity and the expression of CD80 and CD86 and increase the expression of TGF-β1, in immature DCs, thus promoting a low T cell-activating capacity in mature DCs (13). Neutrophil ectosomes contain the enzymes myeloperoxidase, elastase, matrix metalloproteinase 9 and proteinase 3, which suggests that neutrophil-derived ectosomes are “ecto-organelles” with antimicrobial activity against opsonized microorganisms in the extracellular milieu (14). In fact, recent studies showed that neutrophil-derived EVs contain antimicrobial proteins from the neutrophil granules, and that these EVs form integrin-dependent aggregates with *Staphylococcus aureus*, impairing bacterial growth (15). Our group demonstrated for the first time that human neutrophils infected *in vitro* with Mtb H37Rv release EVs with a diameter of 500–1,000 nm, and that these EVs express CD35, phosphatidylserine, gp91Phox, Rab5, Rab7, and a subunit of cytochrome b555 (16). However, the effect of these EVs on other cells that are present at the infected site, such as macrophages, is not completely understood. Therefore, we investigated the effect of EVs derived from Mtb-infected neutrophils on human macrophages. In this study, we characterized the EVs released by non-stimulated human neutrophils (“spontaneous” EVs), and those released by neutrophils stimulated with an activator (PMA), a peptide derived from bacterial proteins (fMLF) or an intracellular pathogen (Mtb), in terms of their size and heterogeneity and their TLR-ligand content. We also evaluated the ability of these different types of EVs to affect cytokine, superoxide anion and NO production, and the expression of costimulatory molecules on macrophages, and determined if the EVs altered the intracellular growth of Mtb and the cellular mechanism involved in this intracellular killing of Mtb.

## Materials and Methods

### Mtb Culture

*Mycobacterium tuberculosis* H37Rv (Mtb) TMC 102 strain was grown in Middlebrook 7H9 (BD BBL, NJ, USA) with 10% glycerol and 10% OADC (BD BBL, NJ, USA) for 4 weeks at 37°C, until the logarithmic phase was reached. Bacteria were harvested by centrifugation and stored in DMEM (Gibco, CA, USA) with 10% FCS (Gibco) at −70°C.

### Preparation of Human Neutrophil and Macrophage Cultures

Peripheral blood was obtained by venipuncture from healthy volunteers, which signed an informed consent form. This study was approved by the Bioethics Committee of ENCB-IPN (CEI-ENCB 114 011/2013). Fifteen milliliters of peripheral blood were collected in tubes with heparin (BD Vacutainer). Neutrophils were separated by gradient centrifugation on Histopaque 1119-Percoll (Sigma-Aldrich, MO, USA), according to Aga et al. (17). All neutrophil cultures had purity and viability of at least 98%. Monocytes were separated by gradient centrifugation on Histopaque 1077 (Sigma-Aldrich). To obtain macrophages, monocytes were resuspended in RPMI (Gibco) with penicillin (100 U/ml), streptomycin (100 µg/ml), and l-glutamine (2 mM), and placed in 24-well culture plates (2 × 10^6^ cells/well) at 37°C and 5% CO_2_ for 2 h. Wells were washed three times with warm RPMI and cultured in RPMI with 10% FCS at 37°C and 5% CO_2_ overnight. GM-CSF (5 ng/ml, PeproTech, NJ, USA) was added on days 1 and 3, and after 7–10 days, macrophage differentiation was confirmed by flow cytometry analysis. The cells were stained with anti-CD14/APC (clone: HCD14), anti-CD11b/PB (clone: 3.9), HLA-DR/FITC (clone: L243), and anti-CD86/PE-Cy7 (clone: IT2.2) (all from BioLegend, CA, USA); macrophages were CD14+ CD11b+ HLA-DR+ CD86+ (data not shown). Data were analyzed on a BD LSR Fortessa with BD FACSDiva software v. 6.0; data were analyzed with FlowJo software v.7.6 (FlowJo LLC, OR, USA). The neutrophils and the macrophages in each experiment were derived from the same donor.

### Production, Concentration, and Characterization of Neutrophil-Derived EVs

Neutrophils (10 × 10^6^ cells/ml in DMEM) were stimulated with 10 nM phorbol 12-myristate 13-acetate (PMA) (Sigma-Aldrich), or with 1 μM *N*-formylmethionyl-leucyl-phenylalanine synthetic peptide (fMLF) (Sigma-Aldrich), or with Mtb at a multiplicity of infection (MOI) of 10 viable bacteria per cell. Neutrophils were incubated at 37°C and 5% CO_2_ for the indicated times. To determine if neutrophil apoptosis is induced under these conditions, apoptosis was evaluated after 30 min of stimulation with PMA, fMLF or Mtb; dexamethasone (100 ng/ml) (Chinoin, Mexico) was used as positive control. Neutrophils were then stained with annexin V/APC (BioLegend) and propidium iodide (BioLegend) and analyzed by flow cytometry. To concentrate EVs from culture supernatants, the supernatants (from neutrophils stimulated with PMA, fMLF, or Mtb, or from non-stimulated neutrophils) were centrifuged, sequentially, at 300 × *g* for 10 min, 2,000 × *g* for 10 min and ultra-centrifuged at 160,000 × *g* for 60 min in an SW40Ti rotor (Beckman Coulter, CA, USA). The concentrated EVs were resuspended in 50 µl of 0.2 µm-filtered PBS. EVs were stored for no more than 24 h at 4°C before performing the experiments. Determination of the protein concentration in EVs: EVs were lysed with 0.2% SDS and analyzed with the micro-bicinchoninic acid method (ThermoFisher Scientific, MA, USA), according to the manufacturer’s protocol. For all the experiments, the EV suspensions were adjusted to 30 µg of protein per ml of 0.2 μm-filtered PBS. *Analysis of EVs by flow cytometry*: neutrophils were incubated for 10 min with CellVue Jade (Polysciences, PA, USA), a dye that binds phospholipids, and washed with 0.2% BSA in 0.2 µm filtered PBS. The released EVs with the different stimuli (30 µg) were incubated with 5 µl of antihuman CD35/PE (clone: E11) (BioLegend), 5 µl of annexin V/PE-Cy7 (BioLegend), or 5 µl of anti-mouse IgG/PE (isotype control, BioLegend) at 4°C for 1 h. The samples were stored at 4°C until acquisition. EVs were acquired on low flow speed, at a rate of less than 50 events per second on a flow cytometer CytoFLEX S (Beckman Coulter). Basal fluorescence was set with 0.2 µm-filtered PBS (to evaluate electronic noise). To set an acceptable forward-scatter (FSC) range suitable for discriminating electronic noise from EVs, we employed Megamix-Plus FSC beads (BioCytex, Marseille, France), which have different sizes (0.1, 0.3, 0.5, and 0.9 µm.) and are recommended for daily standardization for microparticle measurement on the CytoFLEX (18). The threshold was set to limit the analysis to CellVue Jade-positive events. EVs were detected using violet side scatter (VSSC), which has a greater sensitivity to detect small events; FSC and VSSC scales were set in logarithmic mode, with a threshold of 200 arbitrary units for FSC, and the EV gate was set using microbeads (Megamix-Plus). At least 50,000 total events were acquired for each sample, and the data were analyzed with Kaluza Software 1.3v (Beckman Coulter). *Analysis of EVs by nanoparticle tracking analysis (NTA)*: EVs were resuspended in 1 ml of 0.2 µm-filtered PBS, and analyzed in a NanoSight NS 300 (Malvern Instruments Ltd., Malvern, UK). Latex spheres of 100, 200, and 400 nm (Malvern Instruments) were used to calibrate the equipment. Analysis of EVs by transmission electron microscopy (TEM): EVs were obtained as previously described, with an extra centrifugation of 10,000 × *g* for 30 min before ultra-centrifugation at 160,000 × *g* to improve TEM images. After ultra-centrifugation, EVs were resuspended in 0.2 µm-filtered PBS and fixed with 1% glutaraldehyde for 20 min. The sample was then absorbed for 2 min on a nickel mesh grid, previously shaded with polyvinyl formal and carbon. After washing, EVs were stained with 2% uranyl acetate, and the mesh grid was observed in a JEM 1400 electron microscope (JEOL USA Inc., MA, USA).

*Detection of TLR ligands in EVs*: 2 × 10^5^ HEK cells, stably transfected with human TLR2/6, 4, or 5 (InvivoGen, CA, USA), were stimulated with EVs (30 µg of total protein) that were produced by non-stimulated neutrophils (EV-NS), or by neutrophils stimulated with PMA (EV-PMA), fMLF (EV-fMLF), or Mtb (EV-TB) for 30 min. As positive controls, cells were stimulated with Zymosan (InvivoGen, 10 µg/ml) for TLR2/6 activation, lipopolysaccharide (LPS) from *Escherichia coli* O111:B4 (InvivoGen, 10 µg/ml) for TLR4 activation, and flagellin from *Salmonella typhimurium* (InvivoGen, 10 µg/ml) for TLR5 activation. After 24 h, supernatants were collected, and IL-8 was quantified by ELISA (BioLegend), according to the manufacturer’s protocol.

### Cytokine Production and Activation of Macrophages in Response to Neutrophil-Derived EVs

Extracellular vesicles (30 µg total protein/ml), which were produced by non-stimulated neutrophils (EV-NS), or by neutrophils stimulated with PMA (EV-PMA), fMLF (EV-fMLF), or Mtb (EV-TB) for 30 min, were used to stimulate macrophages (2 × 10^5^) for 24 h. As controls, the macrophages were left with medium alone (NS) or were infected with Mtb (2 × 10^6^). After this incubation, the supernatants were collected, centrifuged at 400 × *g* at 4°C and stored at −20°C until analysis. IL-1β, IL-6, IL-10, and TNF-α were measured with a cytometric bead array (BD), and TGF-β was measured with an ELISA Kit (BioLegend), according to the manufacturer’s protocol. In the same experiments, macrophages (detached from the culture plate with cold PBS) were washed and centrifuged at 400 × *g* at 4°C, and stained with anti-CD14/APC, Lin1 (anti-CD3, CD14, CD16, CD19, CD20 and CD56)/FITC, anti-HLA-DR/FITC, anti-CD1a/PE, anti-CD11c/PB, anti-CD80/PE-Cy5, anti-CD86/PE-Cy7, and the corresponding isotype controls (BioLegend), for 15 min at 4°C. Cells were then washed with 1% BSA in PBS and analyzed by flow cytometry.

### Determination of Mtb CFU in Mtb-Infected Macrophages (IM)

Macrophages (2 × 10^5^) were plated in triplicates on 24-well plates, infected with Mtb (2 × 10^6^) for 2 h at 37°C, washed three times with HBSS, and treated with 8 µg/ml amikacin for 2 h (to eliminate extracellular Mtb). Cells were then washed three times with HBSS and stimulated with EVs (30 µg of total protein per ml) for 4 h. Cells were washed with PBS and incubated for 24 or 48 h. The cells were then lysed with 0.2% SDS for 5 min, and the lysis was stopped with 500 µl of 5% albumin. In some experiments, 50 µg/ml rapamycin (Sigma-Aldrich) was added instead of EVs to induce autophagy, and 150 nM wortmannin (Sigma-Aldrich) was added after EV treatment as an autophagy inhibitor.

Intracellular CFU were determined by serial dilutions in PBS, which were plated on Middlebrook-7H10 agar supplemented with glycerol and OADC. Agar plates were incubated at 37°C for 2 weeks. For each time point in each repetition of the experiment, CFU were determined from three different wells.

### Quantification of Superoxide Anion and NO in Mtb-IM

Macrophages (2 × 10^5^) were infected with Mtb (2 × 10^6^) for 2 h. After this incubation, the cells were washed with PBS, treated with 8 µg/ml amikacin for 2 h, and incubated with EVs (EV-NS, EV-PMA, EV-fMLF, or EV-TB) for 4 h. To quantify superoxide anion, cells were washed with PBS and incubated for 15, 30, and 45 min and 1–6 h, in the presence of nitro blue tetrazolium (Sigma-Aldrich), as previously reported (19). NO was quantified using the Griess reagent (Promega, WI, USA), at 1, 2, 4, and 6 h, according to the manufacturer’s protocol. In some cases, NADPH oxidase was inhibited with diphenyliodonium chloride (DPI) (Sigma-Aldrich) before the stimulus with EVs.

### Detection of the Autophagy Marker LC3-II in Macrophages

To determine if EVs induce LC3-II expression, macrophages (2 × 10^5^) were incubated with EVs (EV-NS, EV-PMA, EV-fMLF, or EV-TB) (30 µg/ml) for 4 h. As a positive control for autophagy induction, macrophages were treated with 5 µg/ml of peptidoglycan (Sigma-Aldrich) for 4 h. The cells were then stained with anti-LC3-II (goat anti-MAP LC3 α/β, Santa Cruz Biotechnology, Inc., Midland, ON, Canada) (green) and DAPI (Vector Laboratories, CA, USA) (blue) and examined in a confocal microscope (LSM5 Pascal, Zeiss, Oberkochen, Germany) to determine LC3-II mean fluorescence intensity (MFI). At least 100 cells from each condition were analyzed, and the MFI of LC3-II was calculated using Zeiss LSM image Browser software v.4.2 (Informer Technologies, Inc., Madrid, Spain). To determine if EVs induce the co-localization of LC3-II with Mtb, macrophages (2 × 10^5^) were infected with Mtb (2 × 10^6^) previously stained with CellVue Maroon (Polysciences). After this incubation, the cells were washed with PBS and left untreated (IM), or were incubated with EVs (EV-NS, EV-PMA, EV-fMLF, or EV-TB) for 4 h. The cells were fixed with 4% paraformaldehyde for 20 min at 4°C. Cells were then permeabilized and blocked for 30 min with 4% BSA and 0.25% SDS/Triton X-100, and incubated with primary (goat anti-MAP LC3 α/β) and secondary (donkey anti-goat IgG/FITC, Santa Cruz Biotechnology) antibodies. The slides were mounted with VECTASHIELD with DAPI (Vector Laboratories, CA, USA) and examined in an inverted confocal microscope (LSM5 Pascal, Zeiss). At least 50 cells from each condition were counted, and the percentage of cells with LC3-II+ puncta (autophagosomes) was calculated.

### Ethical Statement

This human study was approved by the Bioethics Committee of Escuela Nacional de Ciencias Biológicas from the Instituto Poltécnico Nacional (CEI-ENCB 011/2013). All written informed consents were received from participants before inclusion in this study.

### Statistical Analysis

Cytokine, superoxide anion, and NO concentrations, and Mtb CFU were compared with ANOVA, followed by Tukey’s test. All other results were compared with Kruskal–Wallis test with Dunn’s posttest. The analysis were performed using GraphPad Prism v. 5.0 (GraphPad Software, CA, USA), and significance was set at *P* < 0.05.

## Results

### Human Neutrophils Produce EVs with Different Physical Characteristics and TLR-Ligand Content in Response to Mtb, PMA and fMLF

Previous studies (20, 21) had reported that monocytes, platelets and endothelial cells release EVs in response to different activators, from bacterial products like LPS to cellular stress. In this study, we compared the EVs produced by non-stimulated human neutrophils (“spontaneous” EVs, EV-NS) to the EVs produced by neutrophils stimulated with an activator (PMA), a peptide derived from bacterial proteins (fMLF) or an intracellular pathogen (Mtb).

**Figure 1:**
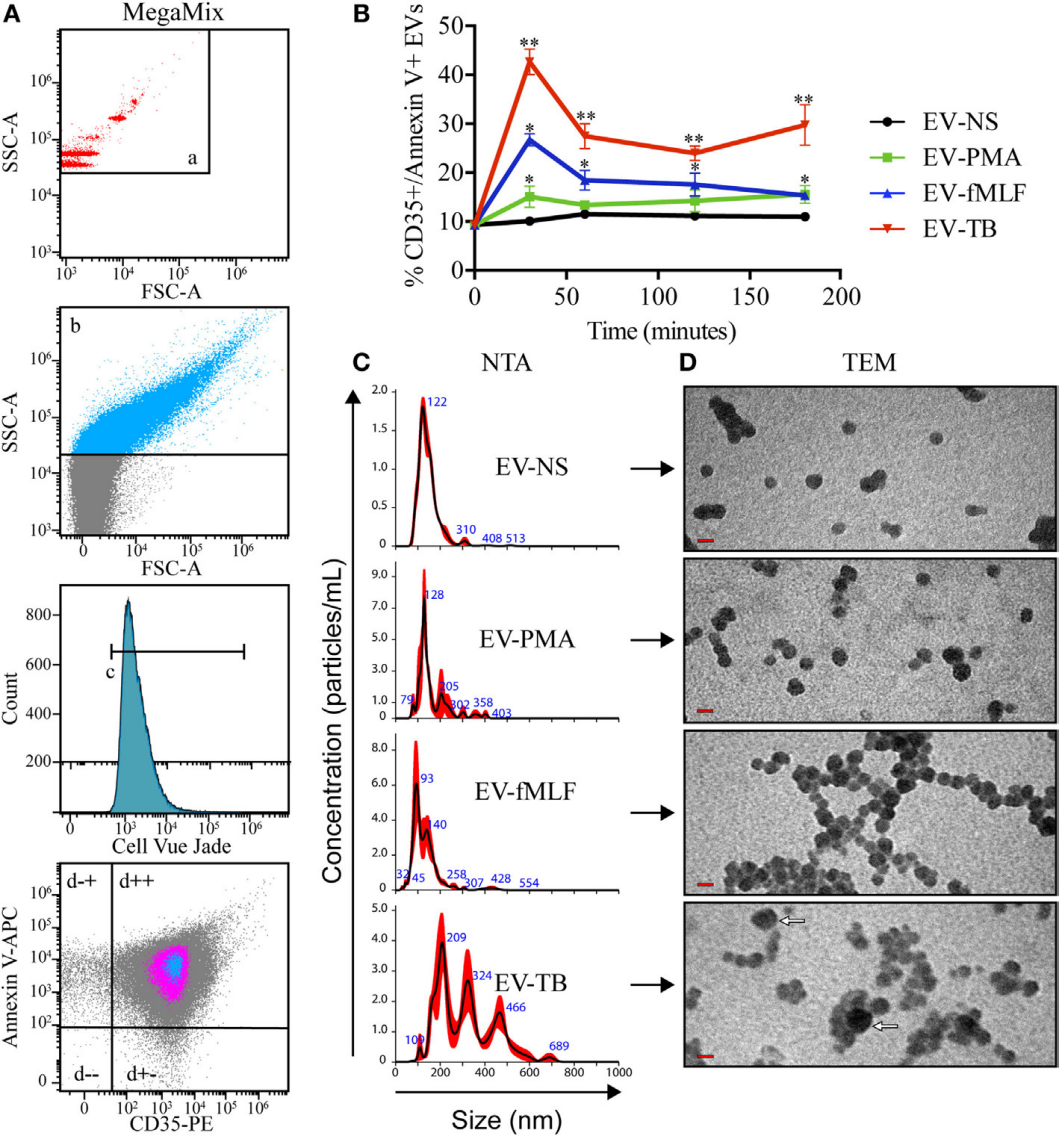
Neutrophil extracellular vesicles (EVs) that are induced by *Mycobacterium tuberculosis* (Mtb) have different characteristics than neutrophil EVs induced by PMA or fMLF. EVs were derived from neutrophils that were left with medium alone (EV-NS) or stimulated with PMA (EV-PMA), fMLF (EV-fMLF), or with Mtb (EV-TB) for the indicated times. **(A)** Gating strategy for the flow cytometry analysis of EVs. (a) Side scatter (SSC-A) of MegaMix^®^ beads of different sizes, which allowed the differentiation of EVs from the electronic noise. (b) SSC-A of neutrophil-derived EVs, (c) EVs derived from neutrophils stained with CellVue Jade, and (d) analysis of the expression of CD35 and annexin V on neutrophil-derived EVs. **(B)** Percentage of CD35+, annexin V+ EVs derived from neutrophils activated with different stimuli. Data points represent mean and SEM from four independent experiments and were analyzed (at each time point) with Kruskal–Wallis test with Dunn’s posttest. Asterisks on the graph represent significant differences between EV-NS and EV-PMA, EV-fMLF, or EV-TB (*P < 0.05 and **P < 0.01). **(C)** Nanoparticle tracking analysis (NTA) of EVs derived from neutrophils activated with different stimuli. Three measurements were made from each sample. A result representative of four independent experiments is shown. **(D)** Transmission electron microscopy (TEM) of EVs derived from neutrophil activated with different stimuli. The red bars indicate 100 nm, and the white arrows indicate the largest EVs. The images are representative of three independent experiments.

Figure 1A shows the flow cytometry analysis of these EVs. The gating strategy includes the calibration of the cytometer with beads of different sizes (a) to allow the differentiation of EVs from the electronic noise, the EVs were acquired in a highly sensitive cytometer for analysis of microvesicles, the EVs were analyzed as single events and CellVue Jade-positive events (c). Since this is a dye that binds phospholipids, the positive events correspond to structures that contain a lipid membrane. The gate in the fourth panel (Figures 1A–D) shows homogenous population of EVs that express CD35 and phosphatidylserine (annexin V+), which have been previously described as markers of ectosomes (21). Figure 1B shows the percentage of CD35+/annexin V+ EVs among EVs that were produced by non-stimulated neutrophils (EV-NS), or by neutrophils stimulated with PMA (EV-PMA), fMLF (EV-fMLF) or Mtb (EV-TB) for the indicated times. At 30 min, the percentage of CD35+/annexin V+ EVs is higher in EV-TB than in EV-NS. EV-TB continued to be more abundant at 180 min; however, at this time point the neutrophils are positive for both annexin V and propidium iodide, and the EVs are likely to contain apoptotic bodies. For all the subsequent experiments, EVs were concentrated from the supernatants of neutrophils stimulated with PMA, fMLF or Mtb for 30 min, when neutrophils are not apoptotic or necrotic (Figure S1 in Supplementary Material).

Figure 1C shows the size distribution of the four types of EVs (EV-NS, EV-PMA, EV-fMLF, and EV-TB), as determined by NTA. EV-TB are more heterogeneous than the other EVs, and most of the vesicles in the EV-TB preparation are larger in diameter than 200 nm (100–700 nm). Most of the vesicles in the EV-NS preparation have a diameter of 100–200 nm, while the vesicles in the EV-PMA preparation have a diameter of 100–300 nm, and those in the EV-fMLF have a diameter of 100–200 nm. EV-TB have only vesicles with higher concentration of particles/ml than EV-NS, EV-PMA, and EV-fMLF (Videos S1–S4 in Supplementary Material). TEM shows dense spheres in the four types of EVs; EV-NS contains the smallest vesicles (50–100 nm), compared with EV-PMA and EV-fMLF, whose vesicles are larger than 200 nm. EV-fMLF contains a larger proportion of aggregated vesicles, compared with other EVs.

**Figure 2:**
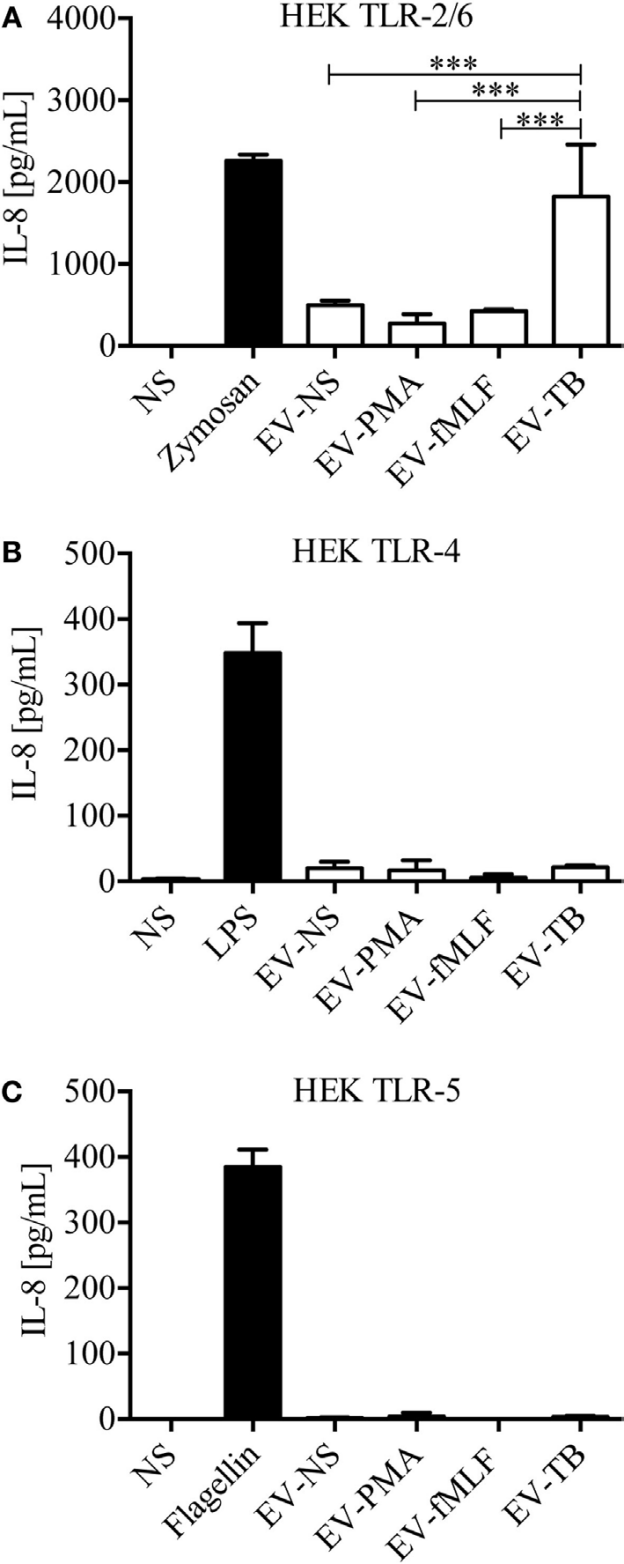
Extracellular vesicles (EVs) derived from *Mycobacterium tuberculosis* (Mtb)-infected neutrophils (EV-TB) contain ligands for toll-like receptors (TLR) 2/6. HEK cells expressing TLR2/6 **(A)**, TLR4 **(B)**, or TLR5 **(C)** were stimulated with EVs that were produced by non-stimulated neutrophils (EV-NS) or by neutrophils stimulated with PMA (EV-PMA), fMLF (EV-fMLF), or Mtb (EV-TB) for 30 min. Zymosan (TLR 2/6), lipopolysaccharide (LPS) (TLR4) and flagellin (TLR5) were used as positive controls, as indicated, and non-stimulated cells were used as negative controls (NS). After 24 h, supernatants were collected, and IL-8 was quantified. Data points represent mean and SD from three independent experiments and were analyzed with one-way ANOVA followed by Tukey’s test (***P < 0.001).

Because we observed a heterogeneity in EVs depending on the stimulus that induced their release, and previous studies have described a difference in the protein content of different EVs, which carry information from the parent cell (22), we tested whether EV-TB contain ligands for TLRs. We observed that EV-TB induced the highest activation of HEK cells stably transfected with human TLR2/6 (Figure 2A). No ligands for TLR4 and 5 were detected in all the EV tested (Figures 2B,C). These results indicate that neutrophils stimulated with Mtb release EVs with intrinsic physical characteristics, which are different from the physical characteristics of EVs induced by different signals, and that EV-TB have the ability to differentially interact with innate immune receptors.

### EV-TB Induce the Production of Pro-inflammatory Cytokines and the Expression of Costimulatory Molecules by Human Macrophages

**Figure 3:**
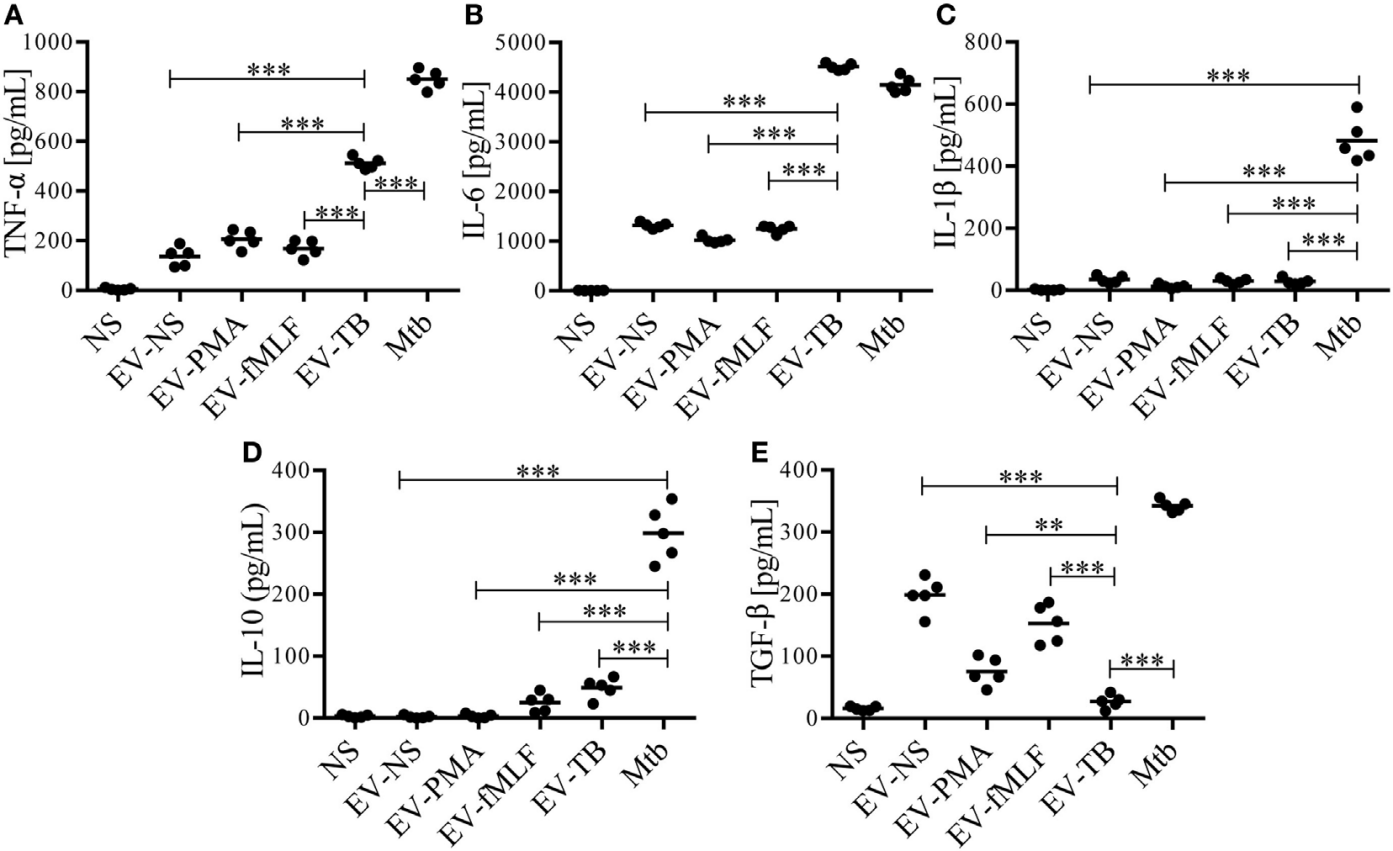
Neutrophil-derived extracellular vesicles (EVs) that are induced by *Mycobacterium tuberculosis* (Mtb) induce the production of TNF-α and IL-6 and IL-10 by macrophages. Macrophages were stimulated for 24 h with EVs that were produced by non-stimulated neutrophils (EV-NS), or by neutrophils stimulated with PMA (EV-PMA), fMLF (EV-fMLF), or Mtb (EV-TB) for 30 min. As controls, the macrophages were left with medium alone (NS) or were infected with Mtb. TNF-α **(A)**, IL-6 **(B)**, IL-1β **(C)**, IL-10 **(D)**, and TGF-β **(E)** were measured in the supernatants. The graphs represent the results obtained with cells from five different healthy volunteers and were analyzed with one-way ANOVA followed by Tukey’s test (*P < 0.05, **P < 0.01, and ***P < 0.001).

**Figure 4:**
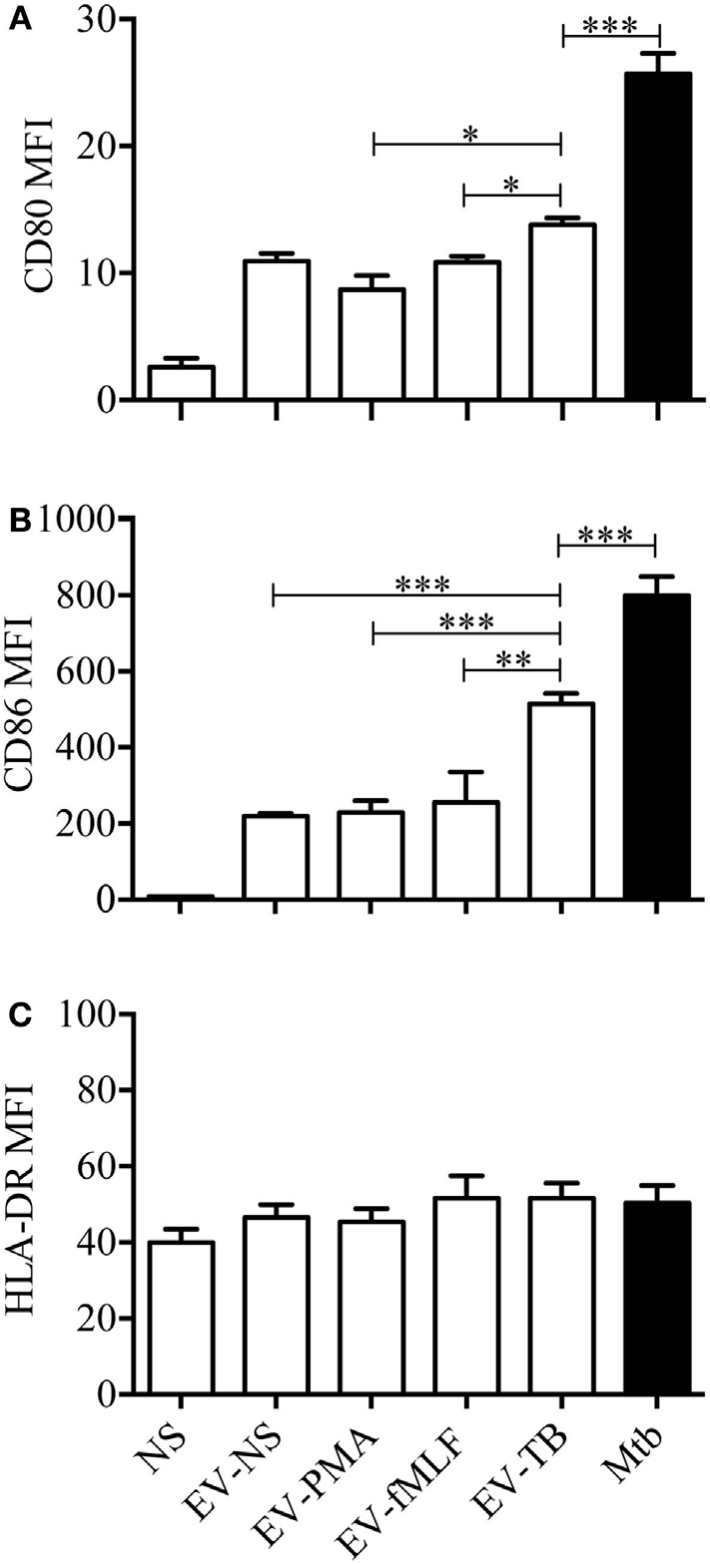
Effect of neutrophil-derived extracellular vesicles (EVs) on the expression of costimulatory molecules in macrophages. Macrophages were stimulated for 24 h with EVs that were produced by non-stimulated neutrophils (EV-NS), or by neutrophils stimulated with PMA (EV-PMA), fMLF (EV-fMLF), or *Mycobacterium tuberculosis* (Mtb) (EV-TB) for 30 min. As controls, the macrophages were left with medium alone (NS) or were infected with Mtb. The expression of CD80 **(A)**, CD86 **(B)**, and HLA-DR **(C)** was analyzed by flow cytometry. The graphs represent the results obtained with cells from five different healthy volunteers, and were analyzed with Kruskal–Wallis test and Dunn’s posttest (*P < 0.05, **P < 0.01, and ***P < 0.001).

Toll-like receptor 2/6 plays an important role in the recognition of mycobacterial lipopeptides by inducing the production of pro-inflammatory cytokines (23), so we decided to evaluate the production of cytokines on macrophages stimulated with EV-NS, EV-PMA, EV-fMLF, or EV-TB for 24 h. As expected, EV-TB induced the highest production of TNF-α, IL-6, and IL-10, and the lowest amounts of TGF-β in macrophages, compared with the other EVs (Figure 3). By contrast, EV-TB were unable to induce IL-1β (Figure 3C). Because TLR activation also leads to an increase in costimulatory molecules and MHC class II proteins in macrophages (24), we tested whether different EVs induced a different expression of these molecules on macrophages. We observed that EV-TB induced the highest expression of the costimulatory molecule CD86 on macrophages, compared with the expression induced by other EVs, while CD80 expression was significantly increased by EV-TB, compared with EV-PMA and EV-fMLF. No changes were observed in the expression levels of HLA-DR (Figure 4). These data indicate that EV-TB differentially regulate macrophage activation, when compared with other neutrophil-derived EVs.

### EV-TB Reduce the Amount of Intracellular Mtb and Increase Superoxide Anion Production and Autophagy in Human Macrophages

Since EV-TB induced the production of pro-inflammatory cytokines and the expression of costimulatory molecules by macrophages, we investigated if these activated macrophages were able to eliminate intracellular Mtb. Macrophages were infected with Mtb for 2 h and then stimulated with EV-NS, EV-PMA, EV-fMLF, or EV-TB and incubated for 24 or 48 h, before CFU determination.

**Figure 5:**
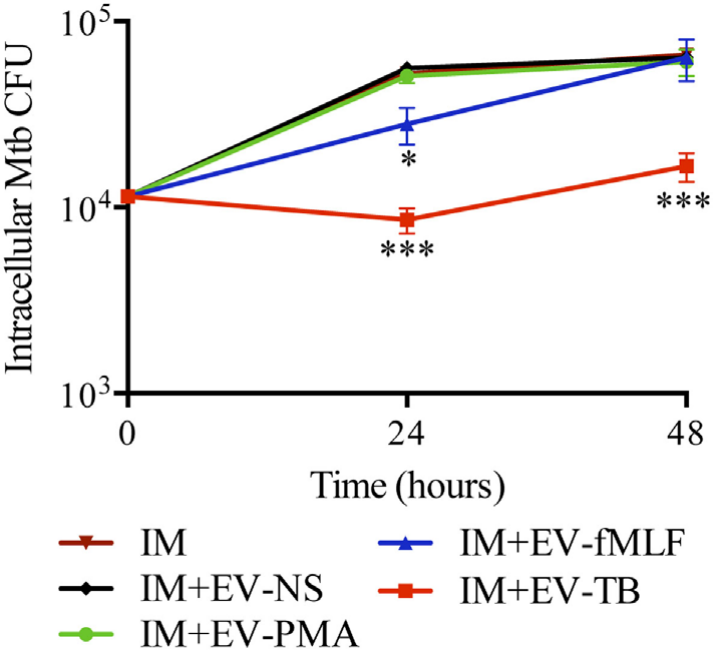
EV-TB reduce the amount of intracellular *Mycobacterium tuberculosis* (Mtb) in macrophages. Macrophages were infected with Mtb at a multiplicity of infection (MOI) of 10 for 2 h. Extracellular bacteria were eliminated with amikacin, and the cells were left untreated (IM) or stimulated with the four types of extracellular vesicles (EVs). The EVs were produced by non-stimulated neutrophils (EV-NS), or by neutrophils stimulated with PMA (EV-PMA), fMLF (EV-fMLF), or Mtb at an MOI of 10 (EV-TB) for 30 min. After 4 h of stimulation with EVs, macrophages were washed with PBS and incubated for a total of 24 and 48 h after infection. Finally, IM were lysed, and intracellular bacteria were evaluated through CFU by performing serial dilutions of macrophage lysates. Data points represent the mean and SD from three independent experiments and were analyzed (at each time point) with one-way ANOVA followed by Tukey’s test. Asterisks on the graph represent significant differences between IM and IM plus each EV (*P < 0.05, **P < 0.01, and ***P < 0.001). Abbreviation: IM, infected macrophages.

Figure 5 shows that EV-TB induced a significant decrease in the amount of intracellular Mtb at 24 and 48 h after EV treatment, compared with the other neutrophil-derived EVs.

**Figure 6:**
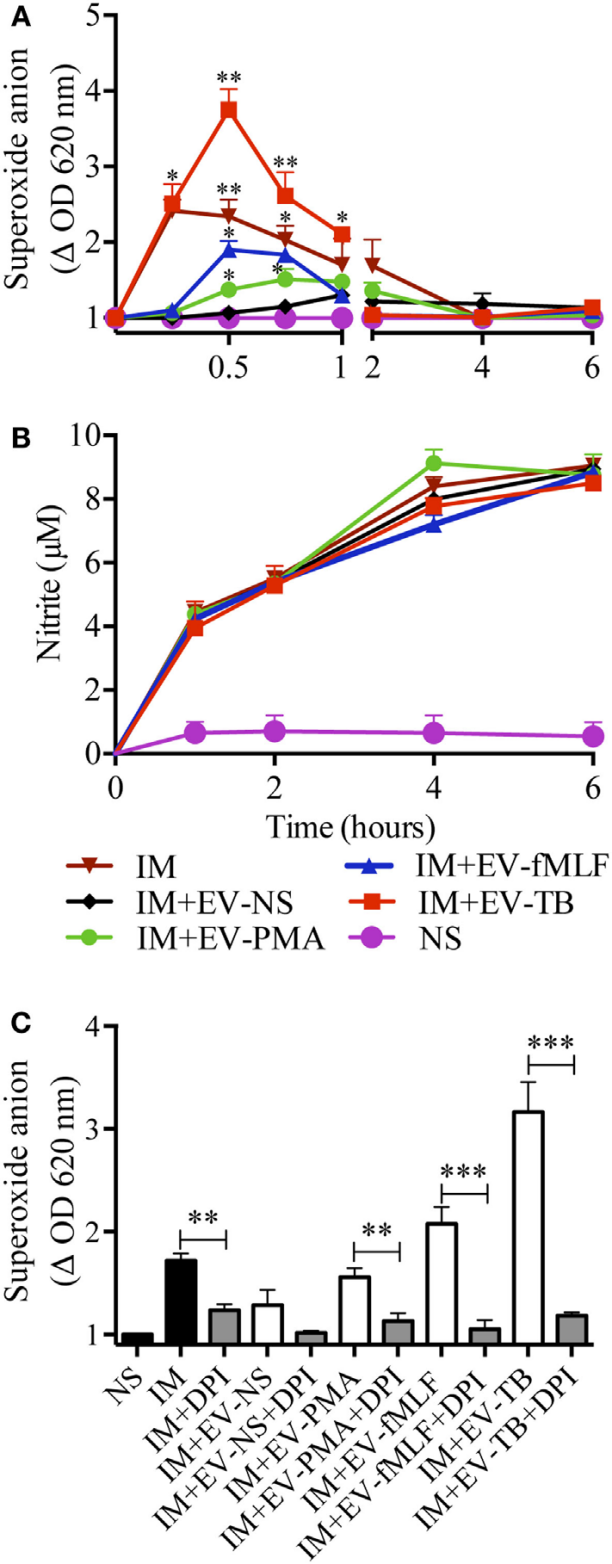
EV-TB induce the production of superoxide anion in *Mycobacterium tuberculosis* (Mtb)-IM. Macrophages were infected with Mtb for 2 h. Extracellular bacteria were eliminated, and macrophages were left untreated (IM), or incubated with extracellular vesicles (EVs) (EV-NS, EV-PMA, EV-fMLF, or EV-TB) for 4 h. Un-IM were used as negative controls (NS). **(A)** Superoxide anion was measured with the nitro blue tetrazolium method. The graph represents the change in optical density at 620 nm ± SD of stimulated cells, compared with untreated cells. **(B)** NO was measured with the Griess method. Data from three independent experiments were analyzed (at each time point) with one-way ANOVA followed by Tukey’s test. Asterisks on the graph represent significant differences between IM and IM plus each EV and the bar in the superoxide anion graph represent significant differences between EV-TB and each EV. **(C)** NADPH oxidase inhibition with DPI. The graph represents the change in optical density at 620 nm ± SD of stimulated cells, compared with untreated cells. Results were obtained with cells from three different healthy volunteers and were analyzed with one-way ANOVA followed by Tukey’s test (*P < 0.05, **P < 0.01, and ***P < 0.001). Abbreviation: IM, infected macrophages.

**Figure 7:**
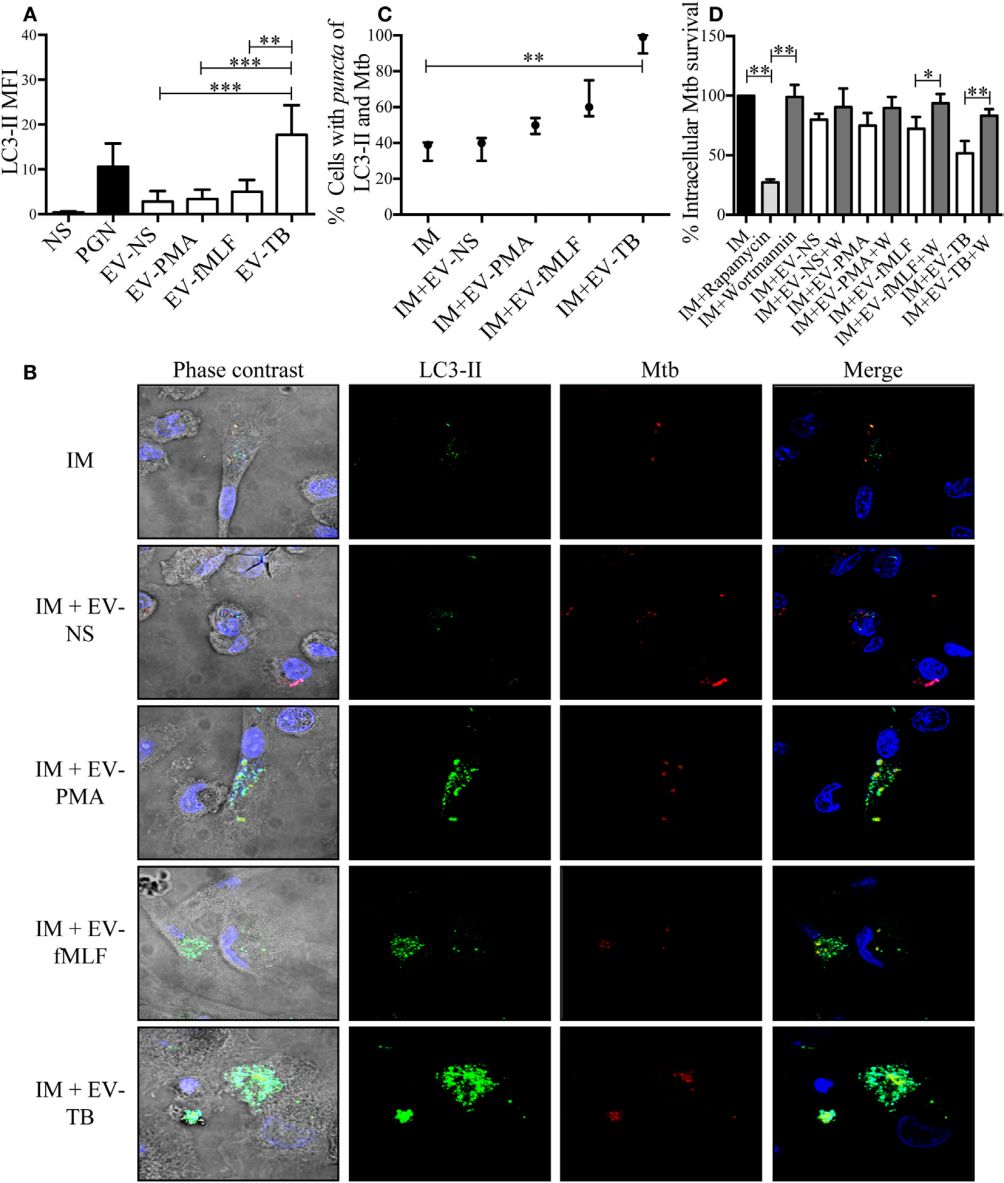
EV-TB-induced autophagy contributes to *Mycobacterium tuberculosis* (Mtb) elimination in Mtb-IM. **(A)** Macrophages were incubated with extracellular vesicles (EVs) (EV-NS, EV-PMA, EV-fMLF, or EV-TB) for 4 h. Non-stimulated cells were used as negative control (NS). As a positive control for autophagy induction, macrophages were treated with peptidoglycan (PGN) for 4 h. The cells were then stained with anti-LC3-II (green) and DAPI (blue) and examined in a confocal microscope to determine LC3-II mean fluorescence intensity (MFI). Data points represent the mean and SEM from three independent experiments and were analyzed with Kruskal–Wallis test and Dunn’s posttest (*P < 0.05 and **P < 0.01). **(B)** Macrophages were infected with Mtb previously stained with CellVue Maroon (red) for 15 min. After this incubation, the cells were left untreated (IM), or were incubated with EVs (EV-NS, EV-PMA, EV-fMLF, or EV-TB) for 4 h. The cells were then stained with anti-LC3-II (green) and DAPI (blue) and examined in a confocal microscope. The images are representative of three independent experiments. **(C)** Percentage of cells with co-localization of LC3-II and Mtb was calculated. The medians with the interquartile ranges are shown (*n* = 3) and were analyzed with Kruskal–Wallis test and Dunn’s posttest (**P < 0.01). **(D)** Percentage of intracellular Mtb survival. Macrophages were infected with Mtb for 2 h. Extracellular bacteria were eliminated, and cells were incubated with EVs (EV-NS, EV-PMA, EV-fMLF, or EV-TB) for 4 h, with or without wortmannin (autophagy inhibitor); IM were also treated with rapamycin (autophagy positive control) or wortmannin. The cells were washed with PBS and incubated for 4 h. The cells were then lysed, and intracellular bacteria were evaluated through CFU by performing serial dilutions of macrophage lysates. The percentage of Mtb survival was calculated for each condition, with Mtb-IM considered as 100%. Data points represent the mean and SEM from three independent experiments and were analyzed with Kruskal–Wallis test and Dunn’s posttest (*P < 0.05, **P < 0.01, and ***P < 0.001). Abbreviation: IM, infected macrophages.

To investigate the mechanism that allowed macrophages to kill intracellular Mtb, we first focused on superoxide anion and NO production, because they have been implied in controlling intracellular Mtb (25, 26). Superoxide anion and NO were measured in Mtb-IM that were treated with the four EVs. EV-TB induced the highest modulation of superoxide anion when compared with the other EVs at 30 min after EV-TB treatment (Figure 6A) this peak could be attributed to the activity of NADPH oxidase, because DPI inhibited this increase in superoxide anion production (Figure 6C). By contrast, neither of the neutrophil-derived EVs modified NO production in the Mtb-IM (Figure 6B). TLR2/6 ligands and reactive oxygen species (ROS) production are well-known signals that induce autophagy (27, 28), and autophagy is a crucial mechanism to inhibit Mtb survival in macrophages (29), so we investigated if EV-TB were efficient at inducing autophagy in macrophages. We found that EV-TB induced the highest LC3-II expression, compared with macrophages treated with other EVs (Figure 7A). Moreover, EV-TB induced the co-localization of the autophagy marker LC3-II with Mtb in macrophages that internalized Mtb (Figures 7B,C), suggesting that autophagy could contribute to EV-TB-induced Mtb elimination. To test this hypothesis, we treated macrophages with wortmannin, an autophagy inhibitor (30), and evaluated intracellular viable Mtb. We observed that Mtb-IM stimulated with EV-TB and treated with this inhibitor showed a full recovery of mycobacterial survival (Figure 7D). By contrast, IM that were treated with rapamycin, an autophagy inductor (31), showed a decreased survival of intracellular Mtb (Figure 7D). Taken as a whole, our results indicate that Mtb activates neutrophils to release EVs with unique physical and biological properties, which are different to those of the EVs induced by other activation pathways in neutrophils. Moreover, EV-TB have the ability to activate macrophages, promoting the control of Mtb intracellular survival through autophagy.

## Discussion

Neutrophils display different effector mechanisms in response to Mtb infection, including phagocytosis and the induction of NETs. In this work, we described another mechanism that is deployed by neutrophils and allows intercellular communication with macrophages through EV release. Previous studies have reported that EVs released from activated neutrophils can regulate the functions of macrophages and DCs (13, 20). In this study, we characterized the EVs that are produced by neutrophils, spontaneously (EV-NS), and in response to PMA (EV-PMA), fMLF (EV-fMLF), or Mtb (EV-TB). All our experiments were performed in an autologous system, which means that the neutrophils used to produce the EVs and the monocyte-derived macrophages were from the same donor. In this system, we detected the release of EVs that express CD35 and phosphatidylserine, which binds annexin V, after neutrophil stimulation with PMA, fMLF or Mtb. However, EV-TB contained a higher percentage of CD35+/annexin V+ EVs after 30 min, compared with EV-NS. These results indicate that vesiculation occurs at the earliest stages of neutrophil activation and it occurs independently from apoptosis, since we did not detect apoptotic neutrophils at this time point. In addition to these CD35+/annexin V+ vesicles, EV-NS, EV-PMA, EV-fMLF, and EV-TB also contained annexin V-vesicles, and previous studies indicate that these vesicles could have an incomplete translocation of phosphatidylserine to the outer layer of their membranes, caused by inactivation of the “scramblase” enzyme (32). The EVs that were released spontaneously from neutrophils (EV-NS) had different physical characteristics than the EVs that were released by activated neutrophils, and different stimuli induced the production of EVs that also differed in their physical characteristics and TLR-ligand content. EV-TB were the most heterogeneous in size, EV-NS were the smallest of the four EVs, and EV-fMLF contained a larger proportion of aggregated vesicles, compared with other EVs, which may indicate that EV-fMLF contain a larger proportion of adhesion molecules, as has been observed previously (33). Mtb is recognized by the innate immune system through several PRRs, including TLR2/1, TLR2/6, TLR4, TLR5, and possibly TLR8, which has been implicated in the recognition of mycobacterial cell wall-associated glycolipids (34). We found no detectable ligands for TLR2/6, TLR4, or TLR5 in EV-NS, EV-PMA, and EV-fMLF, which suggests that these EVs do not carry damage-associated molecular patterns or alarmins that could activate these receptors. EV-TB contained ligands for TLR2/6, which is not unexpected, since several components of Mtb are known TLR2/6 ligands, including lipoarabinomannan (ManLAM), lipomannan, phosphatidylinositol mannoside, and the 19 and 38 kDa lipoproteins (35, 36). This indicates that mycobacterial components reach neutrophil-derived EVs in as little as 30 min after infection; the “sorting” mechanism responsible for this effect has not yet been described. Bhatnagar et al. reported that exosomes derived from Mtb-IM could induce a pro-inflammatory response in macrophages (37); this response could be caused by one or more of the 40 mycobacterial components that are transported in these vesicles, including the TLR2/6 ligand ManLAM (38). However, other studies report that exosomes derived from Mtb-IM have immune-suppressing effects, but these effects are attributed to the miRNAs that are transported in these exosomes, which interfere with the translation of genes involved in cellular activation and inflammation (39).

We observed that EV-TB induced the production of higher amounts of TNF-α and IL-6, and the expression of higher levels of costimulatory molecules in macrophages, compared with the other EVs; these results correlate with a significant decrease in intracellular Mtb CFU in Mtb-IM after EV treatment. TNF-α and IL-6 are well-known macrophage activators, which increase the production of superoxide anion, NO, antimicrobial peptides, and other antimicrobial molecules. In fact, EV-TB induced the production of higher amounts of superoxide anion, with a peak at 30 min after EV-TB treatment, compared with the other EVs. It has been reported that EVs have a direct antimicrobial effect: they can be considered “ecto-organelles” that contain a high concentration of proteolytic enzymes from neutrophils granules (14). Timár et al. reported that neutrophil-derived EVs contain lactoferrin and myeloperoxidase, and directly eliminate *S. aureus*; this bactericidal effect is independent of NET formation (15). However, another study reported that EVs derived from Mtb-infected neutrophils interfere with the antibacterial activity of human macrophages against virulent Mtb (40), in contrast with our results. The difference in outcome could be explained by the differences in the MOI for neutrophil infection, the time allotted for EV release by neutrophils, the amount of EVs used for macrophage activation, the duration of Mtb infection in macrophages and the protocol for obtaining EVs; for instance, Duarte et al. used a different centrifugation protocol, that could lead to the enrichment of different types of EVs.

Autophagy is a highly conserved mechanism that delivers proteins or whole organelles to lysosomes for degradation. The induction of autophagy in Mtb-IM results in increased fusion of Mtb-containing and LC3-II-expressing autophagosomes with lysosomes, which leads to increased bactericidal activity (29). Autophagy can be induced by ROS (27) and also by TLR activation (41). Mycobacterial lipoprotein LpqH induces autophagy through TLR2, and TLR2 stimulation with the mycobacterial lipoprotein LpqH robustly induces antibacterial autophagy through the activation of vitamin D receptor signaling and the induction of cathelicidin synthesis (27). Here, we report that EV-TB induced the expression of the autophagy-related marker LC3-II in macrophages, and the co-localization of LC3-II with Mtb inside these macrophages. These results suggest that autophagy is a key mechanism through which EV-TB reduce intracellular Mtb in macrophages. In fact, we found that blocking autophagy in Mtb-IM (using the autophagy inhibitor wortmannin) increases the survival of Mtb. We detected TLR2/6 ligands in EV-TB, which could be the autophagy inducers in our model. The induction of autophagy inhibits IL-1β secretion by degrading pro-IL-1β (42), and in our experiments, EV-TB were unable to induce a significant amount of IL-1β, which coincides with an increase in LC3-II expression in macrophages. However, Mtb is not completely eliminated from EV-TB treated macrophages; the ability of Mtb to persist inside macrophages is well documented. In particular, Mtb can block autophagosome maturation to create a replication niche; Mtb upregulates miR-155 in an ESAT6-dependent manner to avoid elimination and to promote infection in macrophages (43), and Mtb also induces miR-33 to inhibit autophagy and to reprogram the host lipid metabolism to enable its intracellular survival (44). In conclusion, our study demonstrated that neutrophils produce EVs in response to different activators, and that these EVs differ in their physical characteristics and TLR-ligand content. Furthermore, these EVs can modulate the response of other cells of the innate immune system. In particular, EV-TB activate macrophages and promote the clearance of intracellular Mtb through high production of superoxide anion and autophagy. Whether superoxide anion is enough to confer resistance needs to be clarified in future works. To the best of our knowledge, this phenomenon represents a new mechanism by which neutrophils participate in the control of Mtb infection.

## Ethics Statement

This human study was approved by the Bioethics Committee of Escuela Nacional de Ciencias Biológicas from the Instituto Poltécnico Nacional (CEI-ENCB 011/2013). All written informed consents were received from participants before inclusion in this study.

## Author Contributions

VA-J, KL-P, MG-M, LV-F, VG-P, MC-N, IR-C, VR-G, JC-C, and SG-P performed experiments and analyzed data; VA-J, KL-P, BG-P, VR-G, JM-H, SG-P, and VO-N analyzed and interpreted data; VA-J, CW-B, SE-P, JS-L, IW-B, RC-S, and IE-G interpreted data, drafted the manuscript, and contributed with intellectual content; RC-S and IE-G designed and supervised the study and obtained funding. All the authors critically revised and approved the final version of this manuscript.

## Conflict of Interest Statement

The authors declare that the research was conducted in the absence of any commercial or financial relationships that could be construed as a potential conflict of interest.

## Supplementary Material

Figure S1 Percentage of live neutrophils after 30 and 180 min of treatment with PMA, fMLF, or *Mycobacterium tuberculosis* (Mtb). Neutrophils were left with medium alone or were stimulated with PMA, fMLF, or with Mtb for 30 min **(A)** or for 180 min **(B)**. Neutrophils were stained with annexin V/PE-Cy7 and propidium iodide and analyzed by flow cytometry. The graph represents live (annexin V−/PI−) and apoptotic/dead neutrophils (annexin V+/PI+, annexin V+/PI−, and annexin V−/PI+) for each condition.

Videos S1–S4 EV-TB have higher concentration of particles per milliliter than other extracellular vesicles (EVs). EV-NS, as well as EV-PMA, EV-fMLF, and EV-TB (produced by neutrophils after 30 min) were analyzed by nanoparticle tracking analysis; non-stimulated cells (Video 1), stimulated with PMA (Video 2), fMLF (Video 3), or with *Mycobacterium tuberculosis* (Video 4). The videos show 10 s of Brownian movement from EVs, the data were correlated with particle concentration.
