# Supplementary figures and images for: Extracellular Vesicles Released from Mycobacterium tuberculosis-Infected Neutrophils Promote Macrophage Autophagy and Decrease Intracellular Mycobacterial Survival
Source: Front Immunol. 2018 Feb 19;9:272. doi: 10.3389/fimmu.2018.00272 (PMC5827556; doi:10.3389/fimmu.2018.00272)

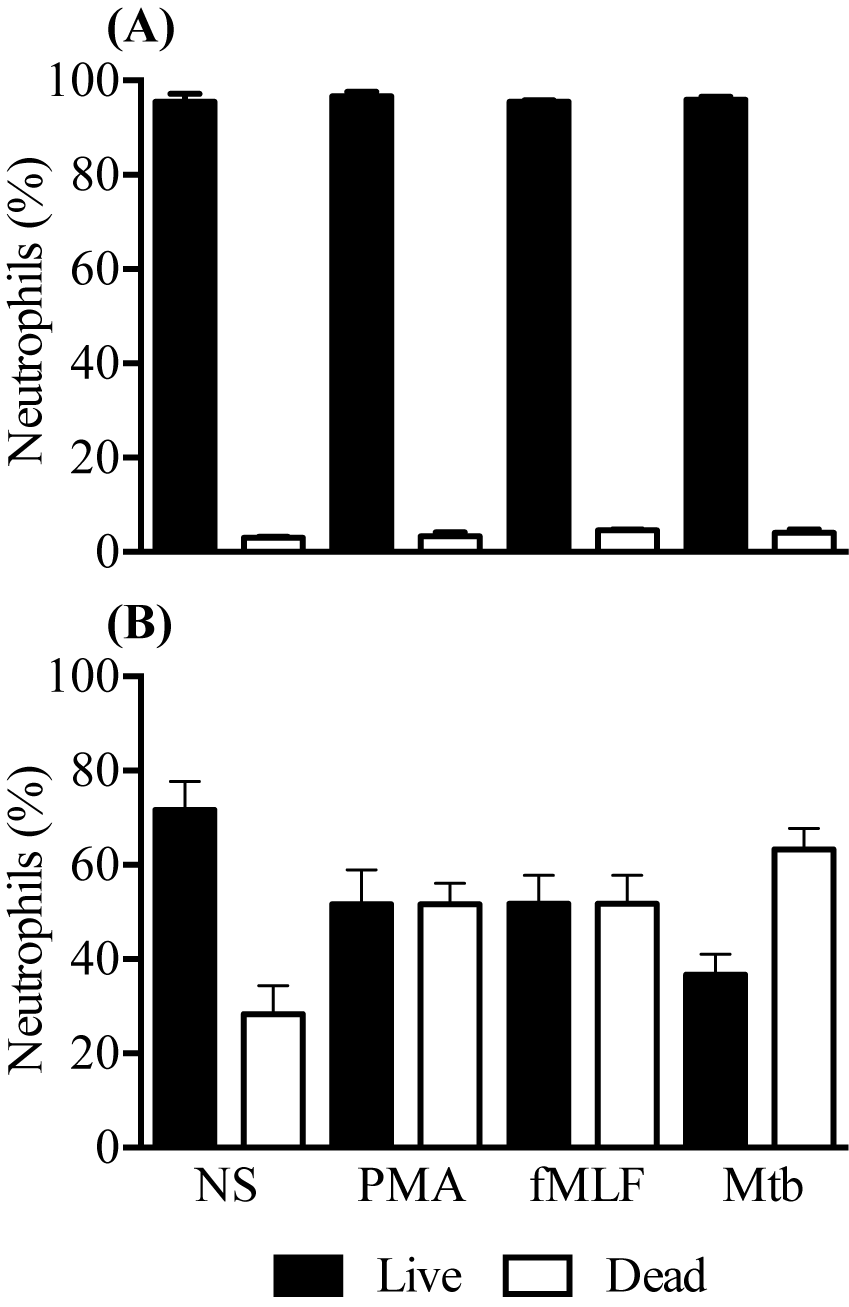

Supplement: Figure S1 — Percentage of live neutrophils after 30 and 180 min of treatment with PMA, fMLF, or Mycobacterium tuberculosis (Mtb). Neutrophils were left with medium alone or were stimulated with PMA, fMLF, or with Mtb for 30 min (A) or for 180 min (B). Neutrophils were stained with annexin V/PE-Cy7 and propidium iodide and analyzed by flow cytometry. The graph represents live (annexin V−/PI−) and apoptotic/dead neutrophils (annexin V+/PI+, annexin V+/PI−, and annexin V−/PI+) for each condition. [file Image_1.tif]
